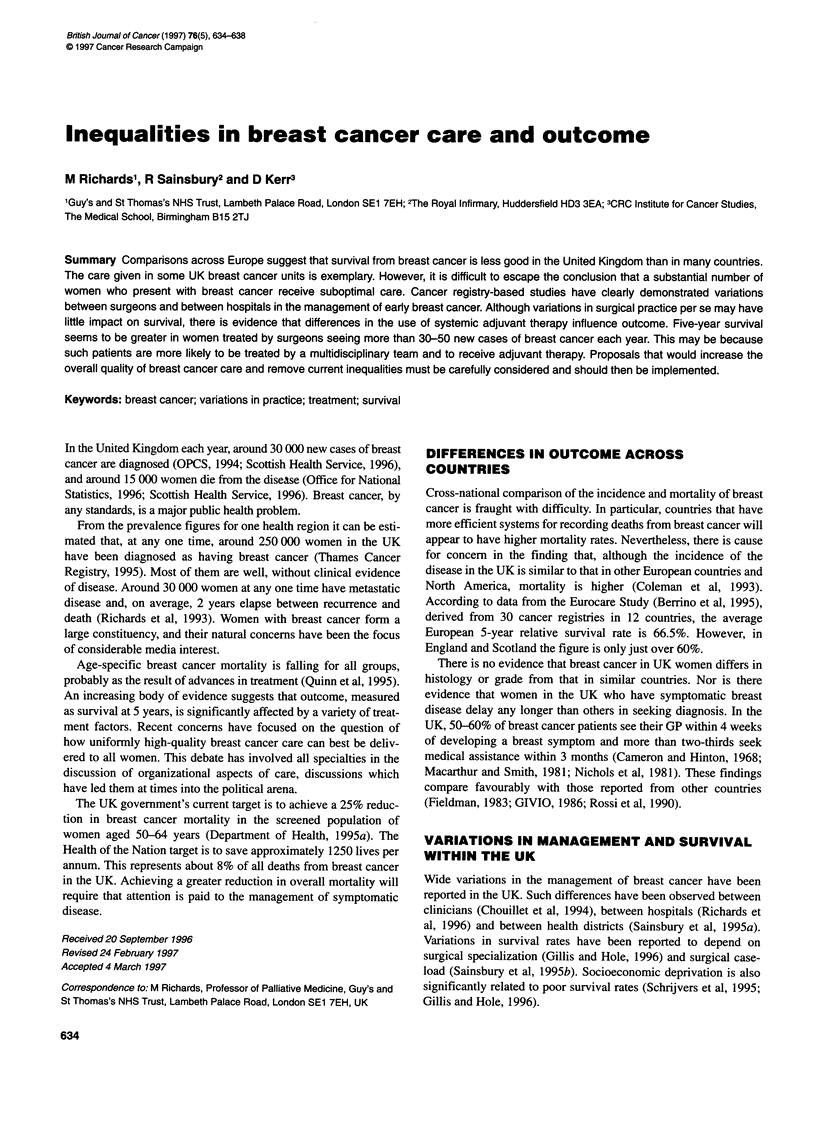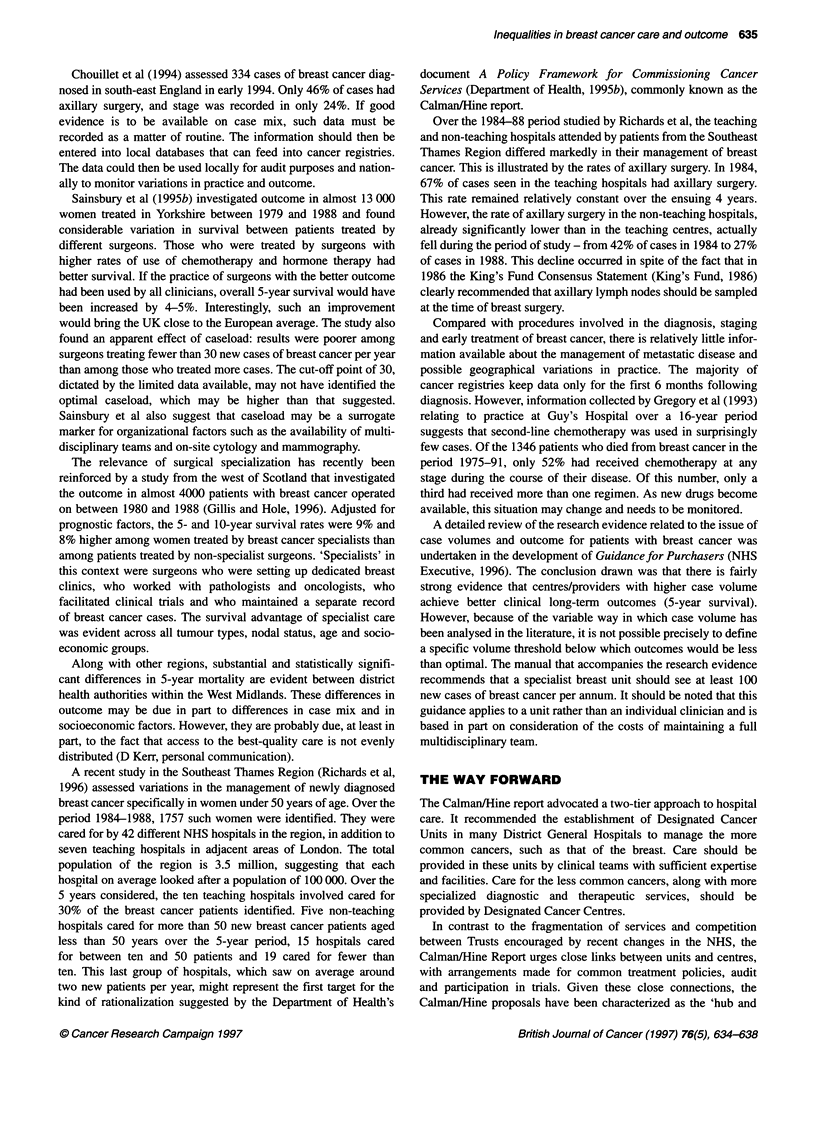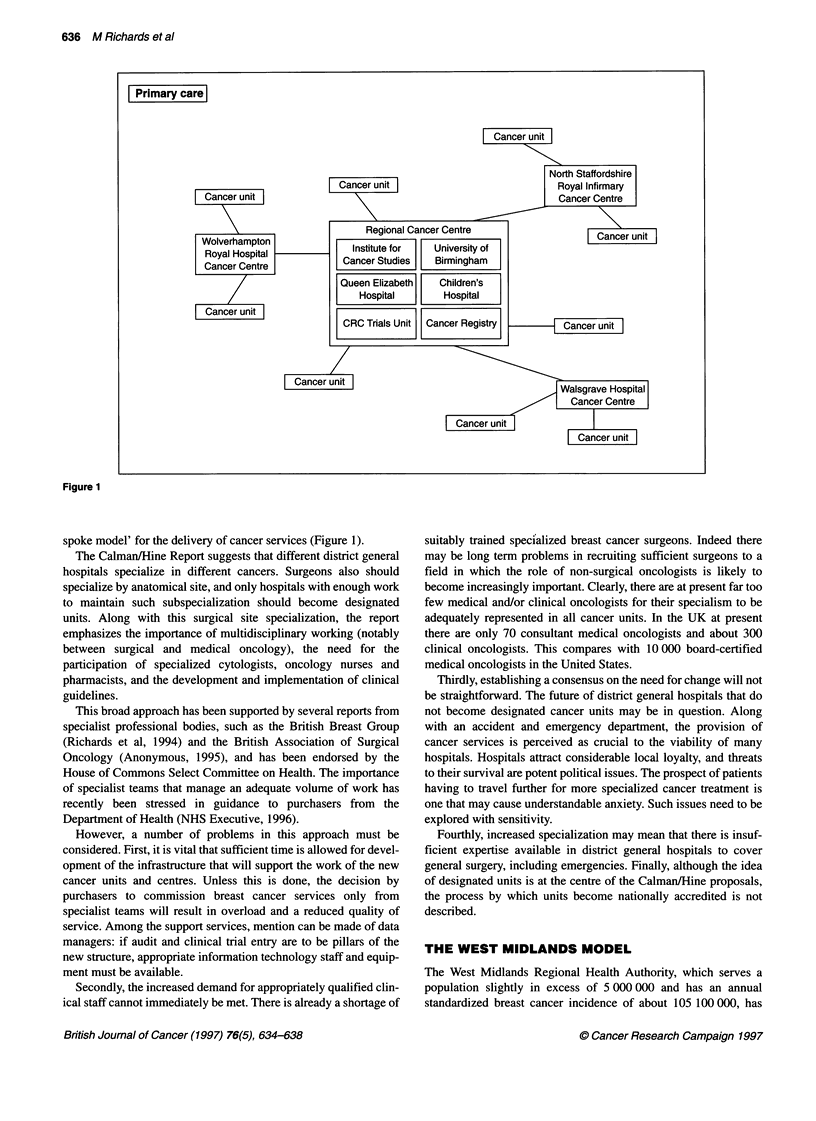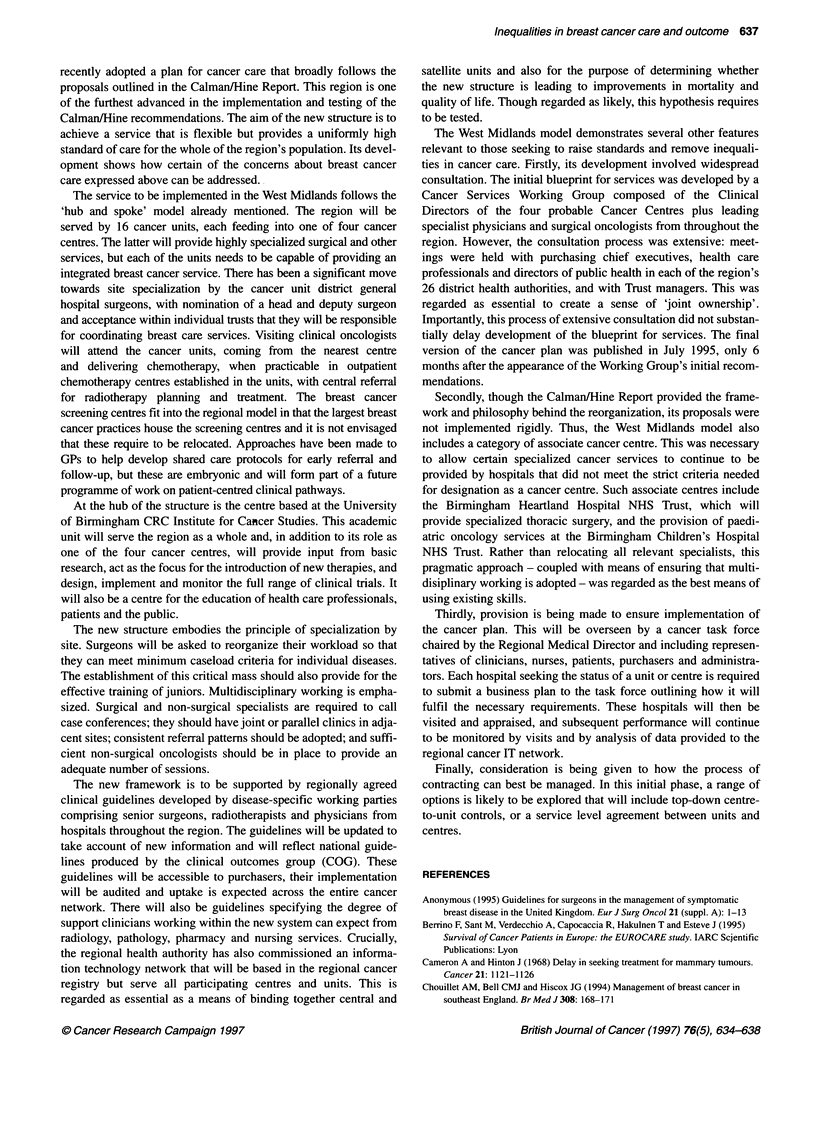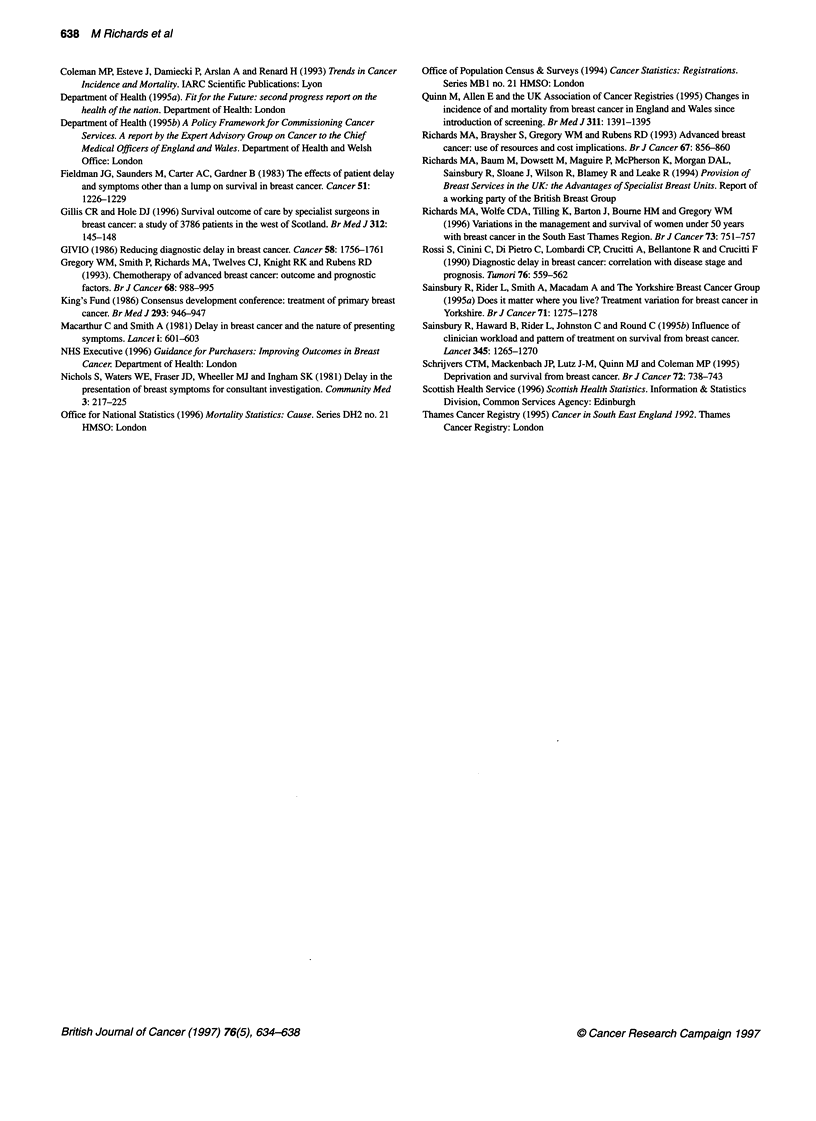# Inequalities in breast cancer care and outcome.

**DOI:** 10.1038/bjc.1997.437

**Published:** 1997

**Authors:** M. Richards, R. Sainsbury, D. Kerr

**Affiliations:** Guy's and St Thomas's NHS Trust, London, UK.

## Abstract

Comparisons across Europe suggest that survival from breast cancer is less good in the United Kingdom than in many countries. The care given in some UK breast cancer units is exemplary. However, it is difficult to escape the conclusion that a substantial number of women who present with breast cancer receive suboptimal care. Cancer registry-based studies have clearly demonstrated variations between surgeons and between hospitals in the management of early breast cancer. Although variations in surgical practice per se may have little impact on survival, there is evidence that differences in the use of systemic adjuvant therapy influence outcome. Five-year survival seems to be greater in women treated by surgeons seeing more than 30-50 new cases of breast cancer each year. This may be because such patients are more likely to be treated by a multidisciplinary team and to receive adjuvant therapy. Proposals that would increase the overall quality of breast cancer care and remove current inequalities must be carefully considered and should then be implemented.


British Journal of Cancer (1997) 76(5), 634–638
© 1997 Cancer Research Campaign

### Inequalities in breast cancer care and outcome

**M Richards[1], R Sainsbury[2] and D Kerr[3]**

[1]Guy's and St Thomas's NHS Trust, Lambeth Palace Road, London SE1 7EH; [2]The Royal Infirmary, Huddersfield HD3 3EA; [3]CRC Institute for Cancer Studies, The Medical School, Birmingham B15 2TJ

**Summary** Comparisons across Europe suggest that survival from breast cancer is less good in the United Kingdom than in many countries. The care given in some UK breast cancer units is exemplary. However, it is difficult to escape the conclusion that a substantial number of women who present with breast cancer receive suboptimal care. Cancer registry-based studies have clearly demonstrated variations between surgeons and between hospitals in the management of early breast cancer. Although variations in surgical practice per se may have little impact on survival, there is evidence that differences in the use of systemic adjuvant therapy influence outcome. Five-year survival seems to be greater in women treated by surgeons seeing more than 30–50 new cases of breast cancer each year. This may be because such patients are more likely to be treated by a multidisciplinary team and to receive adjuvant therapy. Proposals that would increase the overall quality of breast cancer care and remove current inequalities must be carefully considered and should then be implemented.

**Keywords:** breast cancer; variations in practice; treatment; survival

In the United Kingdom each year, around 30 000 new cases of breast cancer are diagnosed (OPCS, 1994; Scottish Health Service, 1996), and around 15 000 women die from the disease (Office for National Statistics, 1996; Scottish Health Service, 1996). Breast cancer, by any standards, is a major public health problem.

From the prevalence figures for one health region it can be estimated that, at any one time, around 250 000 women in the UK have been diagnosed as having breast cancer (Thames Cancer Registry, 1995). Most of them are well, without clinical evidence of disease. Around 30 000 women at any one time have metastatic disease and, on average, 2 years elapse between recurrence and death (Richards et al, 1993). Women with breast cancer form a large constituency, and their natural concerns have been the focus of considerable media interest.

Age-specific breast cancer mortality is falling for all groups, probably as the result of advances in treatment (Quinn et al, 1995). An increasing body of evidence suggests that outcome, measured as survival at 5 years, is significantly affected by a variety of treatment factors. Recent concerns have focused on the question of how uniformly high-quality breast cancer care can best be delivered to all women. This debate has involved all specialties in the discussion of organizational aspects of care, discussions which have led them at times into the political arena.

The UK government's current target is to achieve a 25% reduction in breast cancer mortality in the screened population of women aged 50–64 years (Department of Health, 1995*a*). The Health of the Nation target is to save approximately 1250 lives per annum. This represents about 8% of all deaths from breast cancer in the UK. Achieving a greater reduction in overall mortality will require that attention is paid to the management of symptomatic disease.

*Received 20 September 1996*
*Revised 24 February 1997*
*Accepted 4 March 1997*

*Correspondence to:* M Richards, Professor of Palliative Medicine, Guy's and St Thomas's NHS Trust, Lambeth Palace Road, London SE1 7EH, UK

### DIFFERENCES IN OUTCOME ACROSS COUNTRIES

Cross-national comparison of the incidence and mortality of breast cancer is fraught with difficulty. In particular, countries that have more efficient systems for recording deaths from breast cancer will appear to have higher mortality rates. Nevertheless, there is cause for concern in the finding that, although the incidence of the disease in the UK is similar to that in other European countries and North America, mortality is higher (Coleman et al, 1993). According to data from the Eurocare Study (Berrino et al, 1995), derived from 30 cancer registries in 12 countries, the average European 5-year relative survival rate is 66.5%. However, in England and Scotland the figure is only just over 60%.

There is no evidence that breast cancer in UK women differs in histology or grade from that in similar countries. Nor is there evidence that women in the UK who have symptomatic breast disease delay any longer than others in seeking diagnosis. In the UK, 50–60% of breast cancer patients see their GP within 4 weeks of developing a breast symptom and more than two-thirds seek medical assistance within 3 months (Cameron and Hinton, 1968; Macarthur and Smith, 1981; Nichols et al, 1981). These findings compare favourably with those reported from other countries (Fieldman, 1983; GIVIO, 1986; Rossi et al, 1990).

### VARIATIONS IN MANAGEMENT AND SURVIVAL WITHIN THE UK

Wide variations in the management of breast cancer have been reported in the UK. Such differences have been observed between clinicians (Chouillet et al, 1994), between hospitals (Richards et al, 1996) and between health districts (Sainsbury et al, 1995*a*). Variations in survival rates have been reported to depend on surgical specialization (Gillis and Hole, 1996) and surgical caseload (Sainsbury et al, 1995*b*). Socioeconomic deprivation is also significantly related to poor survival rates (Schrijvers et al, 1995; Gillis and Hole, 1996).

Chouillet et al (1994) assessed 334 cases of breast cancer diagnosed in south-east England in early 1994. Only 46% of cases had axillary surgery, and stage was recorded in only 24%. If good evidence is to be available on case mix, such data must be recorded as a matter of routine. The information should then be entered into local databases that can feed into cancer registries. The data could then be used locally for audit purposes and nationally to monitor variations in practice and outcome.

Sainsbury et al (1995*b*) investigated outcome in almost 13 000 women treated in Yorkshire between 1979 and 1988 and found considerable variation in survival between patients treated by different surgeons. Those who were treated by surgeons with higher rates of use of chemotherapy and hormone therapy had better survival. If the practice of surgeons with the better outcome had been used by all clinicians, overall 5-year survival would have been increased by 4–5%. Interestingly, such an improvement would bring the UK close to the European average. The study also found an apparent effect of caseload: results were poorer among surgeons treating fewer than 30 new cases of breast cancer per year than among those who treated more cases. The cut-off point of 30, dictated by the limited data available, may not have identified the optimal caseload, which may be higher than that suggested. Sainsbury et al also suggest that caseload may be a surrogate marker for organizational factors such as the availability of multidisciplinary teams and on-site cytology and mammography.

The relevance of surgical specialization has recently been reinforced by a study from the west of Scotland that investigated the outcome in almost 4000 patients with breast cancer operated on between 1980 and 1988 (Gillis and Hole, 1996). Adjusted for prognostic factors, the 5- and 10-year survival rates were 9% and 8% higher among women treated by breast cancer specialists than among patients treated by non-specialist surgeons. 'Specialists' in this context were surgeons who were setting up dedicated breast clinics, who worked with pathologists and oncologists, who facilitated clinical trials and who maintained a separate record of breast cancer cases. The survival advantage of specialist care was evident across all tumour types, nodal status, age and socioeconomic groups.

Along with other regions, substantial and statistically significant differences in 5-year mortality are evident between district health authorities within the West Midlands. These differences in outcome may be due in part to differences in case mix and in socioeconomic factors. However, they are probably due, at least in part, to the fact that access to the best-quality care is not evenly distributed (D Kerr, personal communication).

A recent study in the Southeast Thames Region (Richards et al, 1996) assessed variations in the management of newly diagnosed breast cancer specifically in women under 50 years of age. Over the period 1984–1988, 1757 such women were identified. They were cared for by 42 different NHS hospitals in the region, in addition to seven teaching hospitals in adjacent areas of London. The total population of the region is 3.5 million, suggesting that each hospital on average looked after a population of 100 000. Over the 5 years considered, the ten teaching hospitals involved cared for 30% of the breast cancer patients identified. Five non-teaching hospitals cared for more than 50 new breast cancer patients aged less than 50 years over the 5-year period, 15 hospitals cared for between ten and 50 patients and 19 cared for fewer than ten. This last group of hospitals, which saw on average around two new patients per year, might represent the first target for the kind of rationalization suggested by the Department of Health's document *A Policy Framework for Commissioning Cancer Services* (Department of Health, 1995*b*), commonly known as the Calman/Hine report.

Over the 1984–88 period studied by Richards et al, the teaching and non-teaching hospitals attended by patients from the Southeast Thames Region differed markedly in their management of breast cancer. This is illustrated by the rates of axillary surgery. In 1984, 67% of cases seen in the teaching hospitals had axillary surgery. This rate remained relatively constant over the ensuing 4 years. However, the rate of axillary surgery in the non-teaching hospitals, already significantly lower than in the teaching centres, actually fell during the period of study – from 42% of cases in 1984 to 27% of cases in 1988. This decline occurred in spite of the fact that in 1986 the King's Fund Consensus Statement (King's Fund, 1986) clearly recommended that axillary lymph nodes should be sampled at the time of breast surgery.

Compared with procedures involved in the diagnosis, staging and early treatment of breast cancer, there is relatively little information available about the management of metastatic disease and possible geographical variations in practice. The majority of cancer registries keep data only for the first 6 months following diagnosis. However, information collected by Gregory et al (1993) relating to practice at Guy's Hospital over a 16-year period suggests that second-line chemotherapy was used in surprisingly few cases. Of the 1346 patients who died from breast cancer in the period 1975–91, only 52% had received chemotherapy at any stage during the course of their disease. Of this number, only a third had received more than one regimen. As new drugs become available, this situation may change and needs to be monitored.

A detailed review of the research evidence related to the issue of case volumes and outcome for patients with breast cancer was undertaken in the development of *Guidance for Purchasers* (NHS Executive, 1996). The conclusion drawn was that there is fairly strong evidence that centres/providers with higher case volume achieve better clinical long-term outcomes (5-year survival). However, because of the variable way in which case volume has been analysed in the literature, it is not possible precisely to define a specific volume threshold below which outcomes would be less than optimal. The manual that accompanies the research evidence recommends that a specialist breast unit should see at least 100 new cases of breast cancer per annum. It should be noted that this guidance applies to a unit rather than an individual clinician and is based in part on consideration of the costs of maintaining a full multidisciplinary team.

### THE WAY FORWARD

The Calman/Hine report advocated a two-tier approach to hospital care. It recommended the establishment of Designated Cancer Units in many District General Hospitals to manage the more common cancers, such as that of the breast. Care should be provided in these units by clinical teams with sufficient expertise and facilities. Care for the less common cancers, along with more specialized diagnostic and therapeutic services, should be provided by Designated Cancer Centres.

In contrast to the fragmentation of services and competition between Trusts encouraged by recent changes in the NHS, the Calman/Hine Report urges close links between units and centres, with arrangements made for common treatment policies, audit and participation in trials. Given these close connections, the Calman/Hine proposals have been characterized as the 'hub and

© Cancer Research Campaign 1997

**Figure 1**

spoke model' for the delivery of cancer services (Figure 1).

The Calman/Hine Report suggests that different district general hospitals specialize in different cancers. Surgeons also should specialize by anatomical site, and only hospitals with enough work to maintain such subspecialization should become designated units. Along with this surgical site specialization, the report emphasizes the importance of multidisciplinary working (notably between surgical and medical oncology), the need for the participation of specialized cytologists, oncology nurses and pharmacists, and the development and implementation of clinical guidelines.

This broad approach has been supported by several reports from specialist professional bodies, such as the British Breast Group (Richards et al, 1994) and the British Association of Surgical Oncology (Anonymous, 1995), and has been endorsed by the House of Commons Select Committee on Health. The importance of specialist teams that manage an adequate volume of work has recently been stressed in guidance to purchasers from the Department of Health (NHS Executive, 1996).

However, a number of problems in this approach must be considered. First, it is vital that sufficient time is allowed for development of the infrastructure that will support the work of the new cancer units and centres. Unless this is done, the decision by purchasers to commission breast cancer services only from specialist teams will result in overload and a reduced quality of service. Among the support services, mention can be made of data managers: if audit and clinical trial entry are to be pillars of the new structure, appropriate information technology staff and equipment must be available.

Secondly, the increased demand for appropriately qualified clinical staff cannot immediately be met. There is already a shortage of suitably trained specialized breast cancer surgeons. Indeed there may be long term problems in recruiting sufficient surgeons to a field in which the role of non-surgical oncologists is likely to become increasingly important. Clearly, there are at present far too few medical and/or clinical oncologists for their specialism to be adequately represented in all cancer units. In the UK at present there are only 70 consultant medical oncologists and about 300 clinical oncologists. This compares with 10 000 board-certified medical oncologists in the United States.

Thirdly, establishing a consensus on the need for change will not be straightforward. The future of district general hospitals that do not become designated cancer units may be in question. Along with an accident and emergency department, the provision of cancer services is perceived as crucial to the viability of many hospitals. Hospitals attract considerable local loyalty, and threats to their survival are potent political issues. The prospect of patients having to travel further for more specialized cancer treatment is one that may cause understandable anxiety. Such issues need to be explored with sensitivity.

Fourthly, increased specialization may mean that there is insufficient expertise available in district general hospitals to cover general surgery, including emergencies. Finally, although the idea of designated units is at the centre of the Calman/Hine proposals, the process by which units become nationally accredited is not described.

### THE WEST MIDLANDS MODEL

The West Midlands Regional Health Authority, which serves a population slightly in excess of 5 000 000 and has an annual standardized breast cancer incidence of about 105 100 000, has

© Cancer Research Campaign 1997

recently adopted a plan for cancer care that broadly follows the proposals outlined in the Calman/Hine Report. This region is one of the furthest advanced in the implementation and testing of the Calman/Hine recommendations. The aim of the new structure is to achieve a service that is flexible but provides a uniformly high standard of care for the whole of the region's population. Its development shows how certain of the concerns about breast cancer care expressed above can be addressed.

The service to be implemented in the West Midlands follows the 'hub and spoke' model already mentioned. The region will be served by 16 cancer units, each feeding into one of four cancer centres. The latter will provide highly specialized surgical and other services, but each of the units needs to be capable of providing an integrated breast cancer service. There has been a significant move towards site specialization by the cancer unit district general hospital surgeons, with nomination of a head and deputy surgeon and acceptance within individual trusts that they will be responsible for coordinating breast care services. Visiting clinical oncologists will attend the cancer units, coming from the nearest centre and delivering chemotherapy, when practicable in outpatient chemotherapy centres established in the units, with central referral for radiotherapy planning and treatment. The breast cancer screening centres fit into the regional model in that the largest breast cancer practices house the screening centres and it is not envisaged that these require to be relocated. Approaches have been made to GPs to help develop shared care protocols for early referral and follow-up, but these are embryonic and will form part of a future programme of work on patient-centred clinical pathways.

At the hub of the structure is the centre based at the University of Birmingham CRC Institute for Cancer Studies. This academic unit will serve the region as a whole and, in addition to its role as one of the four cancer centres, will provide input from basic research, act as the focus for the introduction of new therapies, and design, implement and monitor the full range of clinical trials. It will also be a centre for the education of health care professionals, patients and the public.

The new structure embodies the principle of specialization by site. Surgeons will be asked to reorganize their workload so that they can meet minimum caseload criteria for individual diseases. The establishment of this critical mass should also provide for the effective training of juniors. Multidisciplinary working is emphasized. Surgical and non-surgical specialists are required to call case conferences; they should have joint or parallel clinics in adjacent sites; consistent referral patterns should be adopted; and sufficient non-surgical oncologists should be in place to provide an adequate number of sessions.

The new framework is to be supported by regionally agreed clinical guidelines developed by disease-specific working parties comprising senior surgeons, radiotherapists and physicians from hospitals throughout the region. The guidelines will be updated to take account of new information and will reflect national guidelines produced by the clinical outcomes group (COG). These guidelines will be accessible to purchasers, their implementation will be audited and uptake is expected across the entire cancer network. There will also be guidelines specifying the degree of support clinicians working within the new system can expect from radiology, pathology, pharmacy and nursing services. Crucially, the regional health authority has also commissioned an information technology network that will be based in the regional cancer registry but serve all participating centres and units. This is regarded as essential as a means of binding together central and satellite units and also for the purpose of determining whether the new structure is leading to improvements in mortality and quality of life. Though regarded as likely, this hypothesis requires to be tested.

The West Midlands model demonstrates several other features relevant to those seeking to raise standards and remove inequalities in cancer care. Firstly, its development involved widespread consultation. The initial blueprint for services was developed by a Cancer Services Working Group composed of the Clinical Directors of the four probable Cancer Centres plus leading specialist physicians and surgical oncologists from throughout the region. However, the consultation process was extensive: meetings were held with purchasing chief executives, health care professionals and directors of public health in each of the region's 26 district health authorities, and with Trust managers. This was regarded as essential to create a sense of 'joint ownership'. Importantly, this process of extensive consultation did not substantially delay development of the blueprint for services. The final version of the cancer plan was published in July 1995, only 6 months after the appearance of the Working Group's initial recommendations.

Secondly, though the Calman/Hine Report provided the framework and philosophy behind the reorganization, its proposals were not implemented rigidly. Thus, the West Midlands model also includes a category of associate cancer centre. This was necessary to allow certain specialized cancer services to continue to be provided by hospitals that did not meet the strict criteria needed for designation as a cancer centre. Such associate centres include the Birmingham Heartland Hospital NHS Trust, which will provide specialized thoracic surgery, and the provision of paediatric oncology services at the Birmingham Children's Hospital NHS Trust. Rather than relocating all relevant specialists, this pragmatic approach – coupled with means of ensuring that multidisiplinary working is adopted – was regarded as the best means of using existing skills.

Thirdly, provision is being made to ensure implementation of the cancer plan. This will be overseen by a cancer task force chaired by the Regional Medical Director and including representatives of clinicians, nurses, patients, purchasers and administrators. Each hospital seeking the status of a unit or centre is required to submit a business plan to the task force outlining how it will fulfil the necessary requirements. These hospitals will then be visited and appraised, and subsequent performance will continue to be monitored by visits and by analysis of data provided to the regional cancer IT network.

Finally, consideration is being given to how the process of contracting can best be managed. In this initial phase, a range of options is likely to be explored that will include top-down centre-to-unit controls, or a service level agreement between units and centres.

### REFERENCES

Anonymous (1995) Guidelines for surgeons in the management of symptomatic breast disease in the United Kingdom. *Eur J Surg Oncol* **21** (suppl. A): 1–13

Berrino F, Sant M, Verdecchio A, Capocaccia R, Hakulnen T and Esteve J (1995) *Survival of Cancer Patients in Europe: the EUROCARE study*. IARC Scientific Publications: Lyon

Cameron A and Hinton J (1968) Delay in seeking treatment for mammary tumours. *Cancer* **21**: 1121–1126

Chouillet AM, Bell CMJ and Hiscox JG (1994) Management of breast cancer in southeast England. *Br Med J* **308**: 168–171

© Cancer Research Campaign 1997

Coleman MP, Esteve J, Damiecki P, Arslan A and Renard H (1993) *Trends in Cancer Incidence and Mortality*. IARC Scientific Publications: Lyon

Department of Health (1995*a*). *Fit for the Future: second progress report on the health of the nation*. Department of Health: London

Department of Health (1995*b*) *A Policy Framework for Commissioning Cancer Services. A report by the Expert Advisory Group on Cancer to the Chief Medical Officers of England and Wales*. Department of Health and Welsh Office: London

Fieldman JG, Saunders M, Carter AC, Gardner B (1983) The effects of patient delay and symptoms other than a lump on survival in breast cancer. *Cancer* **51**: 1226–1229

Gillis CR and Hole DJ (1996) Survival outcome of care by specialist surgeons in breast cancer: a study of 3786 patients in the west of Scotland. *Br Med J* **312**: 145–148

GIVIO (1986) Reducing diagnostic delay in breast cancer. *Cancer* **58**: 1756–1761

Gregory WM, Smith P, Richards MA, Twelves CJ, Knight RK and Rubens RD (1993). Chemotherapy of advanced breast cancer: outcome and prognostic factors. *Br J Cancer* **68**: 988–995

King's Fund (1986) Consensus development conference: treatment of primary breast cancer. *Br Med J* **293**: 946–947

Macarthur C and Smith A (1981) Delay in breast cancer and the nature of presenting symptoms. *Lancet* **i**: 601–603

NHS Executive (1996) *Guidance for Purchasers: Improving Outcomes in Breast Cancer*. Department of Health: London

Nichols S, Waters WE, Fraser JD, Wheeller MJ and Ingham SK (1981) Delay in the presentation of breast symptoms for consultant investigation. *Community Med* **3**: 217–225

Office for National Statistics (1996) *Mortality Statistics: Cause*. Series DH2 no. 21 HMSO: London

Office of Population Census & Surveys (1994) *Cancer Statistics: Registrations*. Series MB1 no. 21 HMSO: London

Quinn M, Allen E and the UK Association of Cancer Registries (1995) Changes in incidence of and mortality from breast cancer in England and Wales since introduction of screening. *Br Med J* **311**: 1391–1395

Richards MA, Braysher S, Gregory WM and Rubens RD (1993) Advanced breast cancer: use of resources and cost implications. *Br J Cancer* **67**: 856–860

Richards MA, Baum M, Dowsett M, Maguire P, McPherson K, Morgan DAL, Sainsbury R, Sloane J, Wilson R, Blamey R and Leake R (1994) *Provision of Breast Services in the UK: the Advantages of Specialist Breast Units*. Report of a working party of the British Breast Group

Richards MA, Wolfe CDA, Tilling K, Barton J, Bourne HM and Gregory WM (1996) Variations in the management and survival of women under 50 years with breast cancer in the South East Thames Region. *Br J Cancer* **73**: 751–757

Rossi S, Cinini C, Di Pietro C, Lombardi CP, Crucitti A, Bellantone R and Crucitti F (1990) Diagnostic delay in breast cancer: correlation with disease stage and prognosis. *Tumori* **76**: 559–562

Sainsbury R, Rider L, Smith A, Macadam A and The Yorkshire Breast Cancer Group (1995*a*) Does it matter where you live? Treatment variation for breast cancer in Yorkshire. *Br J Cancer* **71**: 1275–1278

Sainsbury R, Haward B, Rider L, Johnston C and Round C (1995*b*) Influence of clinician workload and pattern of treatment on survival from breast cancer. *Lancet* **345**: 1265–1270

Schrijvers CTM, Mackenbach JP, Lutz J-M, Quinn MJ and Coleman MP (1995) Deprivation and survival from breast cancer. *Br J Cancer* **72**: 738–743

Scottish Health Service (1996) *Scottish Health Statistics*. Information & Statistics Division, Common Services Agency: Edinburgh

Thames Cancer Registry (1995) *Cancer in South East England 1992*. Thames Cancer Registry: London

© Cancer Research Campaign 1997